# Correction: Facile preparation of a polypyrrole modified Chinese yam peel-based adsorbent: characterization, performance, and application in removal of Congo red dye

**DOI:** 10.1039/d2ra90043b

**Published:** 2022-04-19

**Authors:** Yan Wang, Rongyao Chen, Zijing Dai, Qingcai Yu, Yongmei Miao, Ronghua Xu

**Affiliations:** College of Life and Health Sciences, Anhui Science and Technology University Fengyang 233100 China yanwang0129@126.com

## Abstract

Correction for ‘Facile preparation of a polypyrrole modified Chinese yam peel-based adsorbent: characterization, performance, and application in removal of Congo red dye’ by Yan Wang *et al.*, *RSC Adv.*, 2022, **12**, 9424–9434, https://doi.org/10.1039/D1RA08280A.

The authors regret that in the Acknowledgements section, the fund number for the National College Student Innovation Training Program was incomplete. The correct fund number for the National College Student Innovation Training Program is 202110879021.

The Royal Society of Chemistry apologises for these errors and any consequent inconvenience to authors and readers.

## Supplementary Material

